# The Silent Threat: A Case of Iatrogenic Asymptomatic Aortic Dissection Post Coronary Artery Bypass Grafting

**DOI:** 10.7759/cureus.41035

**Published:** 2023-06-27

**Authors:** Ahmad Mahdi, Abdul Rahman Akkawi, Mahmoud Mahdi, Hussam Farhoud

**Affiliations:** 1 Internal Medicine, University of Kansas School of Medicine-Wichita, Wichita, USA; 2 Cardiology, University of Kansas School of Medicine-Wichita, Wichita, USA

**Keywords:** complication of treatment, adult cardiology, cardiology imaging, asymptomatic aortic dissection, coronary artery bypass graft surgery

## Abstract

Asymptomatic aortic dissection (AD) is a rare but potentially life-threatening complication that can occur following coronary artery bypass graft (CABG) surgery. While CABG is a well-established surgical procedure for managing multivessel coronary artery disease, it can inadvertently predispose patients to the development of AD, especially in those with pre-existing aortic pathology. The pathophysiology underlying AD after CABG is multifactorial, with factors, such as atherosclerosis, manipulation of the aorta during surgery, and hemodynamic stress, playing significant roles. Notably, the absence of symptoms poses a diagnostic challenge, as patients may remain unaware of the underlying condition until a catastrophic event occurs. Therefore, a high index of suspicion and vigilant postoperative monitoring are crucial in identifying asymptomatic AD. Diagnostic modalities including imaging techniques, such as computed tomography angiography (CTA), magnetic resonance imaging (MRI), and echocardiography, play pivotal roles in confirming the diagnosis and determining the extent of the dissection. Prompt surgical intervention is generally recommended in symptomatic patients or those with evidence of impending complications. We hereby present a case report of a patient who presented with asymptomatic AD post CABG surgery and discuss the pathophysiology, presentation, diagnostic workup, and treatment options.

## Introduction

Aortic dissection (AD) is a cardiovascular emergency that results from the tearing of the intimal layer of the aorta, leading to the formation of a false lumen within the media. It can lead to a life-threatening condition by compromising blood flow to vital organs, causing rupture, or organ damage [1]. Several risk factors have been associated with an increased incidence of AD. These include hypertension, connective tissue disorders such as Marfan syndrome and Loeys-Dietz syndrome, bicuspid aortic valve, advanced age, male gender, atherosclerosis, pregnancy, cocaine use, and traumatic injury to the chest or aorta [2]. The clinical presentation of AD can vary depending on the location, extent, and propagation of the dissection. The most common symptom is sudden, severe chest or back pain, often described as tearing or ripping in nature [3]. Other symptoms may include shortness of breath, sweating, nausea, vomiting, dizziness, and loss of consciousness. In some cases, AD may be asymptomatic, presenting as an incidental finding on imaging studies [3].

Asymptomatic AD is relatively rare, with estimates suggesting that it accounts for less than 10% of all cases of AD. The prevalence of asymptomatic AD is difficult to determine because the condition is often identified incidentally during diagnostic imaging studies performed for other reasons [4]. Thus, it is important to identify patients at risk and monitor asymptomatic AD to prevent complications, such as aortic rupture or organ malperfusion [5]. Moreover, the occurrence of AD following coronary artery bypass grafting (CABG) is infrequent but has the potential to be a life-threatening complication [6]. When the ascending aorta is used as the site of arterial cannulation, iatrogenic AD (iAD) occurs in approximately 0.06% of cases. However, this figure increases to approximately 0.6% when the femoral or iliac arteries are utilized, and approximately 0.5% when the axillary or subclavian arteries are employed [7]. We hereby present a case of asymptomatic AD in a patient after undergoing CABG and discuss different types of AD, symptomatology, diagnosis, and treatment.

## Case presentation

We report the case of a 77-year-old man with a medical history of Parkinson's disease, hypertension (HTN), atrial fibrillation (AF) status post pacemaker placement (PPM), and heart failure with a preserved ejection fraction that was diagnosed with extensive three-vessel disease by heart catheterization. He subsequently underwent CABG. The patient was heparinized and cannulated for cardiopulmonary bypass. The aorta was cannulated through a double-purse-string suture just proximal to the innominate artery. A single two-stage venous cannula was used, introduced through a purse-string suture in the right atrial appendage. The atrial appendage was palpated, and the right atrial pacing electrode was in the base of the appendage adherent to the free wall. This was carefully avoided during cannulation. Afterward, cardiopulmonary bypass was begun, and the patient was cooled to 34°C. A catheter for retrograde cardioplegia infusion was placed through purse-string suture in the right atrial free wall and directed into the coronary sinus. A catheter for antegrade cardioplegia infusion and ascending aortic root venting was placed through purse-string suture in the ascending aorta. Bypass with a left internal mammary artery (LIMA) to the left anterior descending (LAD) artery, saphenous vein graft to the ramus intermedius, and reverse saphenous vein graft to the obtuse marginal coronary artery with left atrial appendage ligation was performed successfully.

**Figure 1:**
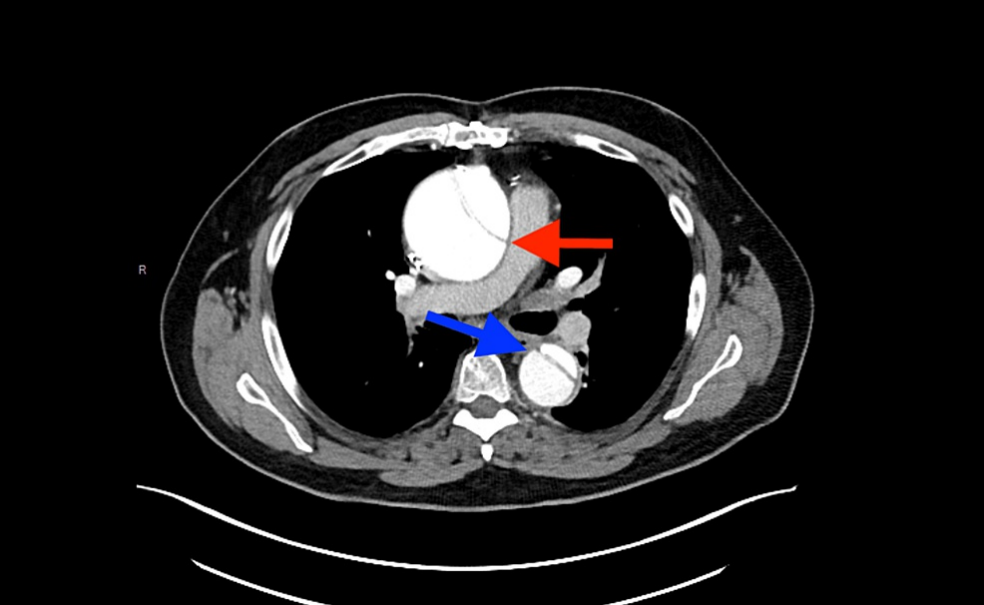
CTA of the chest and abdomen revealing a dissection of the ascending (blue) and descending (red) aorta CTA: computed tomography angiography

**Figure 2:**
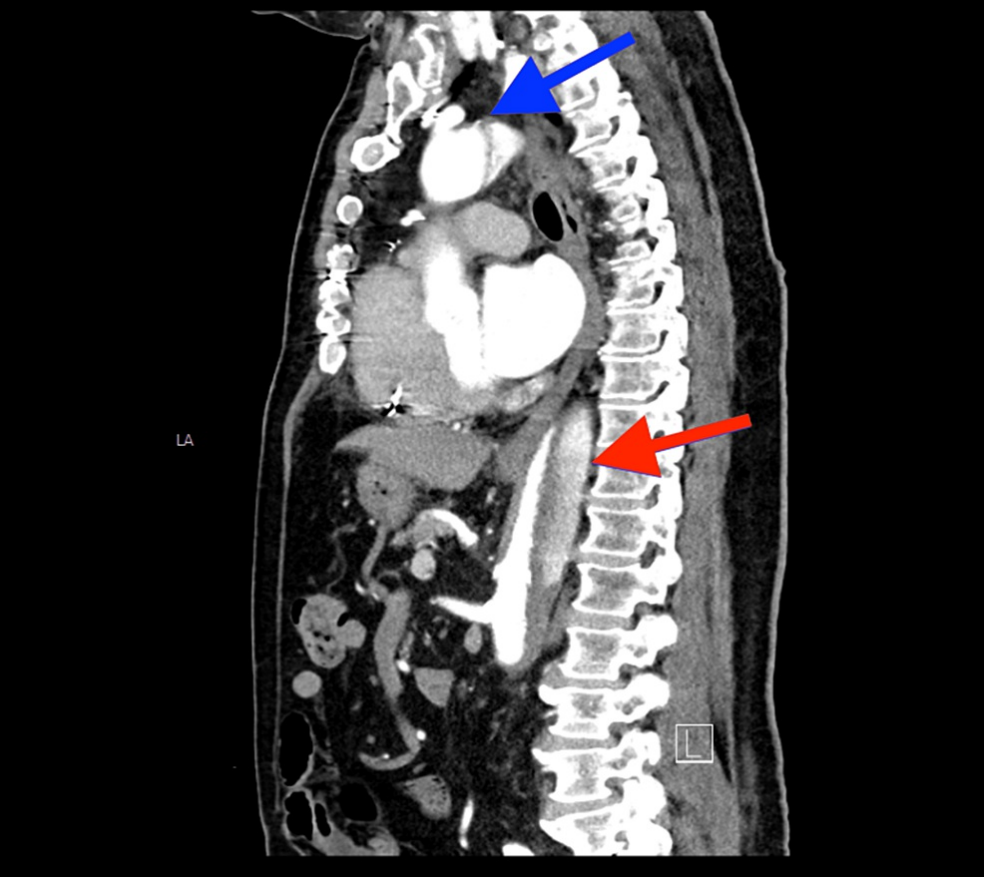
CTA of the chest and abdomen revealing dissection of the ascending (blue) and descending (red) aorta CTA: computed tomography angiography

Two days post-operatively, the patient complained of progressive shortness of breath but denied chest discomfort. Transesophageal echocardiography (TEE) was subsequently performed and showed left ventricular (LV) systolic dysfunction with an ejection fraction of 45-50%, aortic and mitral valve sclerosis, mild mitral and tricuspid insufficiency, and moderate to severe aortic insufficiency. The TEE results also revealed an incidental dissection in the ascending aorta and proximal transverse aortic arch, which were not significantly dilated. The patient underwent computed tomography angiography (CTA) of the chest, which showed extensive dissection in the ascending aorta with a diameter measuring 7 cm and extending to the aortic arch and down to the renal arteries (Figure 1 and Figure 2). This finding was surprising as the patient denied any sudden onset of chest discomfort. His only issue was progressive shortness of breath, and he denied any paroxysmal nocturnal dyspnea (PND) or orthopnea. Due to the significant increase in the size of the aorta, the patient required further evaluation by an aortic surgeon. He underwent aortic valve replacement with a 23 mm Intuity valve. Resection and replacement of the ascending aorta and proximal transverse aortic arch with a tube graft (28 mm woven Dacron tube graft) modified hypothermic circulatory arrest and bilateral antegrade perfusion. The patient's dyspnea resolved, and he was stable post-operatively. He was discharged in a stable condition. A week later, TEE was repeated, showing healed AD with flow in true and false lumens.

## Discussion

Several factors have been proposed as possible contributors to iAD, including an enlarged ascending aorta (greater than 40 mm), a fragile or thin-walled ascending aorta, atherosclerosis affecting the aorta or other arteries used for cannulation, the presence of a bicuspid aortic valve, aortic stenosis, aortic insufficiency, and connective tissue disorders, such as cystic medial necrosis (CMN). In addition, hypertension is often observed in the majority of patients with iAD and is commonly cited as a risk factor particularly during the cannulation and clamping of the aorta [7].

The classification of AD is essential for the accurate diagnosis and treatment of the condition. There are several classification systems for AD, including the Stanford classification and DeBakey classification [3]. The Stanford classification is a widely used classification system that divides AD into two types: Stanford type A and Stanford type B. This classification is based on the location of the dissection in the aorta and the involvement of the ascending aorta. Stanford type A AD involves the ascending aorta and may extend into the aortic arch or descending aorta. Stanford type B AD involves the descending aorta only and does not involve the ascending aorta or aortic arch [8]. The DeBakey classification is another commonly used classification system that divides AD into three types: DeBakey type I, DeBakey type II, and DeBakey type III. This classification is based on the location of the dissection in the aorta and the extent of the involvement. DeBakey type I AD involves the ascending aorta, aortic arch, and descending aorta. DeBakey type II AD involves only the ascending aorta. DeBakey type III AD involves only the descending aorta [9].

The diagnosis of AD requires a high index of suspicion and the use of advanced diagnostic techniques. The clinical presentation of AD can vary widely and can include chest pain, back pain, and neurological symptoms. However, up to 10% of patients may have atypical presentations, such as palpitations, abdominal pain, or neurological deficit, or be asymptomatic [3,4]. In our patient, he presented with shortness of breath, a presentation that has rarely been reported in the literature [10]. A thorough medical history should be obtained, including any known risk factors for AD. According to some studies, AD in heart failure patients is more likely to be asymptomatic and Stanford dissection type A [11]. AD can cause a wide range of physical examination findings, including a difference in blood pressure between the arms, a pulse deficit, or a new murmur. Aortic regurgitation, pericardial effusion, or tamponade may be present in patients with associated complications. In fact, AR is present in 44% of type A dissections and 12% of type B dissections [12]. However, physical examination findings may be normal in up to 20% of patients with AD [13]. Transthoracic echocardiography is a non-invasive test that uses ultrasound waves to produce images of the heart and aorta. This test can detect AD and identify associated complications, such as aortic regurgitation or pericardial effusion. However, TEE has higher sensitivity and specificity, particularly in the detection of ascending AD [14]. Aortic angiography is another invasive testing that aids in AD diagnosis when the above measures are inconclusive [13,15]. Elevated levels of troponin and B-type natriuretic peptide (BNP) may indicate associated myocardial ischemia or dysfunction [16].

Medical therapy is the first-line treatment for patients with uncomplicated type B AD or type A AD who are not surgical candidates. The goals of medical therapy are to lower blood pressure and reduce the shear stress on the aortic wall to prevent further expansion or rupture of the dissection [17]. Endovascular repair is a minimally invasive procedure that involves the placement of a stent graft within the aorta to seal off the entry tear and prevent further expansion or rupture of the dissection. Endovascular repair is the preferred treatment for type B AD with complicated or refractory hypertension and for select cases of type A AD, such as those with suitable anatomy and low surgical risk [18]. Surgical intervention is reserved for patients with type A AD and complicated type B AD or for patients who are not suitable candidates for endovascular repair. Surgical intervention involves the replacement of the diseased segment of the aorta with a synthetic graft. The surgical approach may be open or minimally invasive, depending on the location and extent of the dissection [19].

## Conclusions

Asymptomatic AD is a serious and often life-threatening condition that can go undetected for extended periods. Early diagnosis and prompt management are crucial to prevent adverse outcomes. Therefore, individuals with risk factors for asymptomatic AD should undergo regular monitoring to ensure early detection and management of the condition.
